# Low-dose etoposide-treatment induces endoreplication and cell death accompanied by cytoskeletal alterations in A549 cells: Does the response involve senescence? The possible role of vimentin

**DOI:** 10.1186/1475-2867-13-9

**Published:** 2013-02-05

**Authors:** Anna Litwiniec, Lidia Gackowska, Anna Helmin-Basa, Agnieszka Żuryń, Alina Grzanka

**Affiliations:** 1 Laboratory of Biotechnology, Department of Genetics and Breeding of Root Crops, Plant Breeding and Acclimatization Institute - National Research Institute Radzików, 05-870 Błonie, Research Division in Bydgoszcz, Powstańców Wielkopolskich 10, 85-090, Bydgoszcz, Poland; 2 Laboratory of Clinical and Experimental Immunology, Department of Immunology, Ludwik Rydygier Collegium Medicum in Bydgoszcz, Nicolaus Copernicus University in Toruń, Curie Skłodowskiej 9, 85-094, Bydgoszcz, Poland; 3 Department of Histology and Embryology, Ludwik Rydygier Collegium Medicum in Bydgoszcz, Nicolaus Copernicus University in Toruń, Karłowicza 24, 85-092, Bydgoszcz, Poland

**Keywords:** Senescence-like phenotype, Etoposide, Polyploidy, Depolyploidization, Vimentin, G-actin

## Abstract

**Background:**

Senescence in the population of cells is often described as a program of restricted proliferative capacity, which is manifested by broad morphological and biochemical changes including a metabolic shift towards an autophagic-like response and a genotoxic-stress related induction of polyploidy. Concomitantly, the cell cycle progression of a senescent cell is believed to be irreversibly arrested. Recent reports suggest that this phenomenon may have an influence on the therapeutic outcome of anticancer treatment. The aim of this study was to verify the possible involvement of this program in the response to the treatment of the A549 cell population with low doses of etoposide, as well as to describe accompanying cytoskeletal alterations.

**Methods:**

After treatment with etoposide, selected biochemical and morphological parameters were examined, including: the activity of senescence-associated ß-galactosidase, SAHF formation, cell cycle progression, the induction of p21^Cip1/Waf1/Sdi1^ and cyclin D1, DNA strand breaks, the disruption of cell membrane asymmetry/integrity and ultrastructural alterations. Vimentin and G-actin cytoskeleton was evaluated both cytometrically and microscopically.

**Results and conclusions:**

Etoposide induced a senescence-like phenotype in the population of A549 cells. Morphological alterations were nevertheless not directly coupled with other senescence markers including a stable cell cycle arrest, SAHF formation or p21^Cip1/Waf1/Sdi1^ induction. Instead, a polyploid, TUNEL-positive fraction of cells visibly grew in number. Also upregulation of cyclin D1 was observed. Here we present preliminary evidence, based on microscopic analyses, that suggest a possible role of vimentin in nuclear alterations accompanying polyploidization-depolyploidization events following genotoxic insults.

## Background

Etoposide is a semi-synthetic derivative of podophyllotoxin originating from *Podophyllum peltatum* or *Podophyllum emodi*[1]. This compound acts on topoisomerase II, an enzyme involved in DNA processing during its replication, transcription and recombination. A reversible cleavage of one or both DNA stands by the enzyme is crucial for modulation of its topology, i.e. superhelical structure, as well as for entangling of the molecule [2]. Etoposide affects cleavable enzyme-DNA complexes, by stabilizing them, and thus inhibiting the ability of topoisomerase to ligate these discontinuities [3]. Interaction of such structures with replication forks, transcriptional complexes or other DNA-processing enzymes may finally result in the formation of irreversible DNA strand breaks [4].

This mode of action is known to be related to the major chemotherapeutic effect of the agent, i.e. cell death induction. However, there have also been some suggestions that the response of cancer cells to etoposide also involves other mechanisms, such as chromosomal aberrations, aneuploidy and endoreplication [5,6]. The significance of these phenomena has not yet been fully determined and is currently under discussion with regard to mitotic catastrophe, senescence, neosis and cell survival.

The aim of this paper is to evaluate the influence of low-dose treatment with etoposide on the A549 cell line in the context of some cytoskeletal changes related to the induction of cell death/senescence.

The A549 cell line, which was our experimental model, has been classified as a non-small cell lung carcinoma, adenocarcinoma and was previously derived from type II pneumocytes [7]. As this type of lung cancer is one of the leading causes of death globally, the line is frequently used in studies investigating the effects of different drugs. However, before interpretation of the obtained results, it is crucial to take into consideration the genetic background of the line, i.e. homozygous deletions of *p16*^*Ink4a*^*, p14*^*Arf*^ and *p15*^*Ink4b*^[8-11]. The products of these genes influence the outcome of chemotherapy, due to their participation in tumor suppression via cell death/irreversible cell cycle arrest. Therefore it seems important to evaluate both the possibility and the extent of induction of these different processes in A549 cells.

Some cytoskeletal components have previously been shown to be useful as indicators of cell well-being, and have been described as markers indicative of particular phenomena [12-15]. In this work, we would like to refer to selected alterations in the organization as well as the level of cytoskeletal proteins, especially G-actin and vimentin, defined as potential markers of cellular senescence, in order to perform a more in-depth analysis of the characteristics of cellular response to etoposide.

## Results

### Morphological and biochemical features of senescence/cell death - selected parameters

The influence of etoposide on A549 cells was evaluated based on the manifestation of particular senescence- and/or cell death-related events in the cell population, including SA ß-galactosidase activity, senescence-associated heterochromatin foci (SAHF) formation, p21^Cip1/Waf1/Sdi1^ and cyclin D1 induction, DNA fragmentation rate, as well as the percentage of cells with phosphatidylserine externalization and/or disruptions in the cell membrane. Further insight was gained from transmission electron microscopic observations of the cell ultrastructure and form flow cytometric analysis of cell cycle distribution.

**Figure 1:**
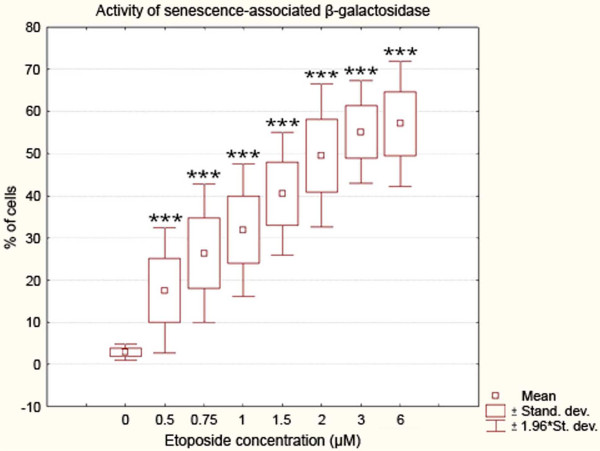
**Activity of senescence-associated ß-galactosidase in A549 cells after treatment with etoposide - statistical data from light microscopic examinations.** Incubation with increasing concentrations of the drug (0.5, 0.75, 1, 1.5, 2, 3 and 6 μM etoposide) for 72 h was followed by further 24 h of incubation in fresh medium. The activity of the enzyme was detected at pH 6. Very highly statistically significant differences as compared to the control cells are marked by *asterisks* (Cochran–Cox statistics, P<0.001). Results are representative of ten independent experiments.

The range of doses used in the initial part of this study was as follows: 0.5; 0.75; 1; 1,5; 2; 3; 6 μM. All these concentrations induced a statistically significant increase in the mean percentage of cells presenting an enhanced activity of SA ß-galactosidase as compared to the control sample (Cochran–Cox statistics; P<0.001) (Figure 1). For further procedures, however, only those concentrations were chosen that caused high and significantly different increases in this activity from one another (Duncan statistics; P<0.05), i.e. 0.75, 1.5 and 3 μM etoposide.

**Figure 2:**
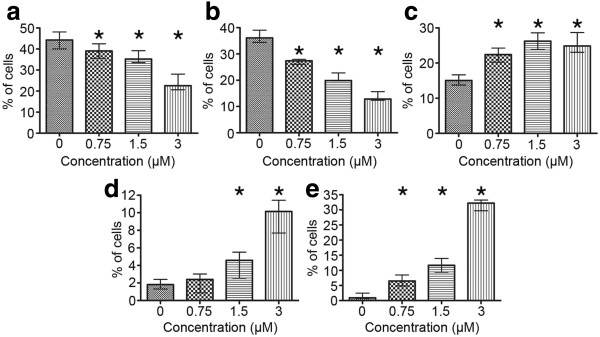
**a-e Cell cycle distribution of A549 cells after treatment with etoposide - statistical data from flow cytometric analyses (staining with RNase/PI). a** Cells with DNA content typical of G_0_/G_1_ phases of the cell cycle, **b** cells with DNA content typical of S phase, **c** cells with DNA content typical of G_2_/M phases, **d** cells with DNA content typical of subG_1_ fraction, **e** cells with DNA content typical of polyploidy. Statistically significant differences as compared to the control cells are marked by *asterisks* (Mann–Whitney U statistics, P<0.05). *Columns* - median percentage of cells, *bars* - interquartile range. Results are representative of five independent experiments.

**Figure 3:**
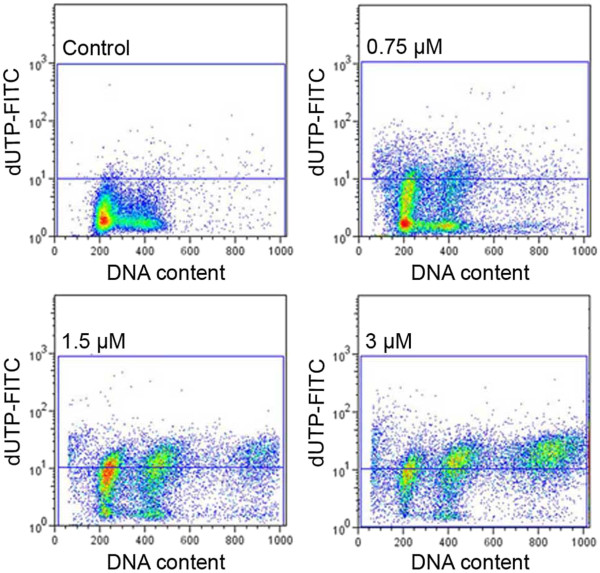
**DNA fragmentation rate in relation to DNA content in A549 cells after treatment with etoposide - representative dot-plots from flow cytometric analyses (the TUNEL method); *upper left *- control cells, *upper right *- 0.75 μM etoposide, *lower left *- 1.5 μM etoposide, *lower right *- 3 μM etoposide.**

All the concentrations of etoposide used in this study (0.75, 1.5, 3 μM) induced a statistically significant decrease in the percentage of A549 cells with DNA content corresponding to G_0_/G_1_ and S in comparison to the control cells (Mann–Whitney U statistics) (Figure 2). Concomitantly, there were statistically significant increases in the population of cells respective to G_2_/M phases and higher DNA contents (>G_2_) (Figure 2). A biparametric analysis of DNA fragmentation rate in relation to its content, resulting from the TUNEL method, revealed that the most significantly damaged fraction was represented by the cells in G_2_/M and, particularly, with higher DNA contents, which was evident at 1.5 and 3 μM etoposide (Figure 3).

**Figure 4:**
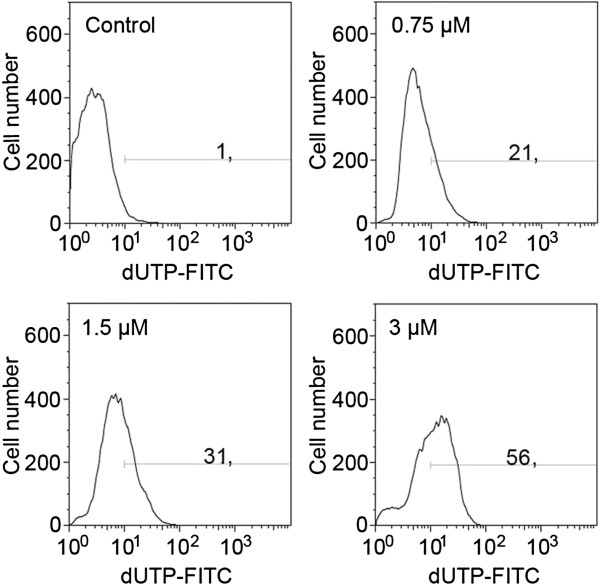
**DNA fragmentation rate in A549 cells after treatment with etoposide - representative histograms from flow cytometric analyses (the TUNEL method); *upper left *- control cells, *upper right *- 0.75 μM etoposide, *lower left *- 1.5 μM etoposide, *lower right *- 3 μM etoposide.**

**Figure 5:**
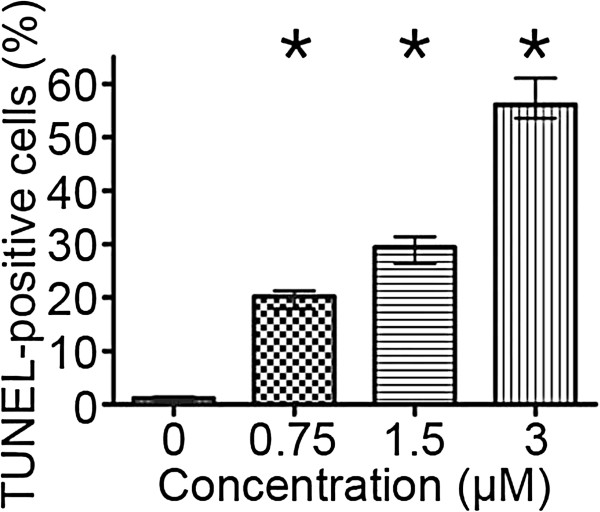
**DNA fragmentation rate in A549 cells after treatment with etoposide - statistical data from flow cytometric analyses (the TUNEL method).** Statistically significant differences as compared to the control cells are marked by *asterisks* (Mann–Whitney U statistics, P<0.05). *Columns* - median percentage of cells, *bars* - interquartile range. Results are representative of five independent experiments.

**Figure 6:**
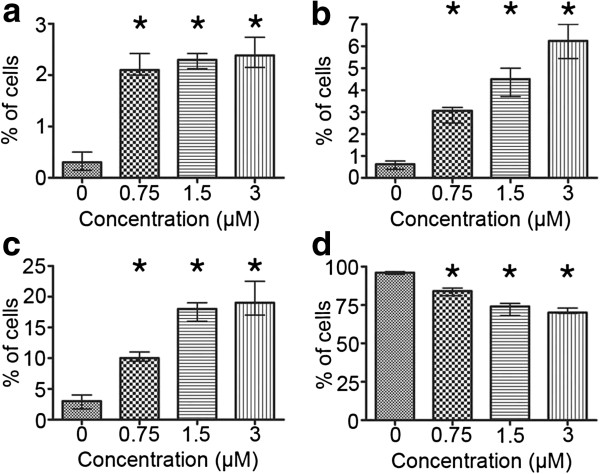
**a-d Cell viability in the population of A549 cells after treatment with etoposide - statistical data from flow cytometric analyses (the annexin V/PI assay).** Cells presenting features of **a** early apoptosis (annexin V+/PI-), **b** late apoptosis (annexin V-/PI-), **c** necrosis (annexin V-/PI+) and **d** viable cells (annexin V-/PI-). Statistically significant differences as compared to the control cells are marked by *asterisks* (Mann–Whitney U statistics, P<0.05). *Columns* - median percentage of cells, *bars* - interquartile range. Results are representative of five independent experiments.

The highest dose of the drug (3 μM) was most effective in the induction of DNA strand breaks, which were present in more than 50% of the cell population (Figure 4). There were statistically significant increases in the number of dUTP-FITC-incorporating cells even after treatment with the lowest of the applied concentrations (0.75 μM) (Figure 5). On the other hand, subG_1_ fraction, respective to cells with DNA content lower than typical of G_0_/G_1_, was visibly increased only in the cells exposed to 1.5 and 3 μM etoposide (Figure 2d). As regards annexin V/PI assay, the results obtained were similar to TUNEL indications, showing statistically significant increases in the percentage of cells manifesting features of early and late apoptosis, as well as necrosis after treatment with all etoposide concentrations used in the study, as compared to the untreated cell population. Concomitantly, the population of cells with preserved integrity and asymmetry of membranes was on the decrease (Figure 6). At the highest dose of etoposide, which turned out to be most effective in cell death induction, the following median values were obtained: 2.6% for annexin V-positive/PI-negative cells (features of early apoptosis), 6.2% for annexin V/PI-positive cells (features of late apoptosis/necrosis) and 19% for annexin V-negative/PI-positive cells (necrotic phenotype).

**Figure 7:**
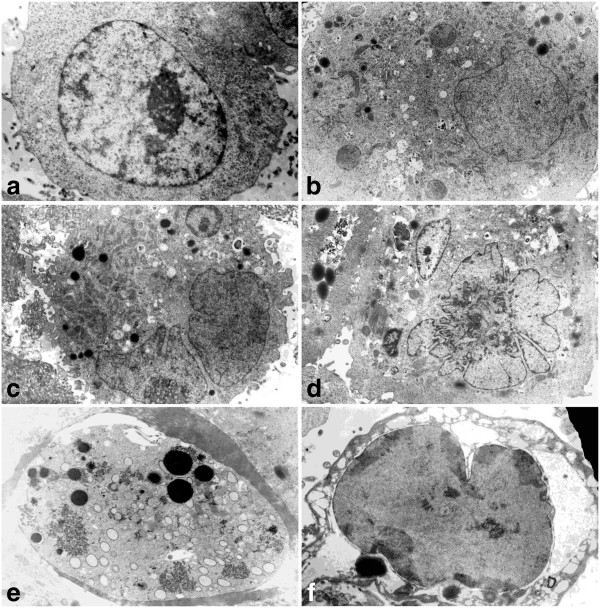
**a-f Morphological and ultrastructural changes in A549 cells after treatment with etoposide - electron microscopic examination. a** Control cells (x 7 000), **b** 0.75 μM etoposide (x 5 000), **c**, **d** 1.5 μM etoposide (x 4 000), **e**, **f** 3 μM etoposide (x 6 000). Progressive alterations in the nucleus and cytoplasm area are evident. At the highest concentration some cells displayed features of extensive lysis/demolition of the cellular content, including dispersed chromatin-like structures appearing in the cytoplasm. Results are representative of five independent experiments.

These biochemical changes were also accompanied by ultrastructural alterations progressing gradually with increasing concentrations. There were some enlarged cells, in which not only cytoplasmic, but also nuclear compartments were visibly affected. Heterogeneity of the cytoplasm manifested itself in the appearance of electron-dense granular structures, including those myelin-like, and electron-clear vacuole-like components (Figure 7). Some of these most probably contained residual materials, while others resembled autophagosomes. Among other organelles, abundant swollen mitochondria, as well as a few dilated endoplasmic reticulum cisternae were present. Visible morphological changes were also related to the nucleus area, and they include an increase in size, chromatin condensations, segmented/lobulated nuclei, as well as multinucleated cells. Besides this, some more pronounced signs typical of cell death may be observed, especially at the highest applied concentration, including highly condensed, densely packed chromatin, extensive cytoplasmic vacuolization, and loss of easily recognizable forms of organelles, suggesting late apoptosis or secondary necrosis (Figure 7e,f). Some cells representing acute damage manifestations, with degenerating cytoplasm, may respond directly by executing necrosis (Figure 7f). Nevertheless, a subpopulation of cells did not show significant morphological differences in comparison with the control group, possibly representing chemotherapy-resistant clones of A549 cells.

**Figure 8:**
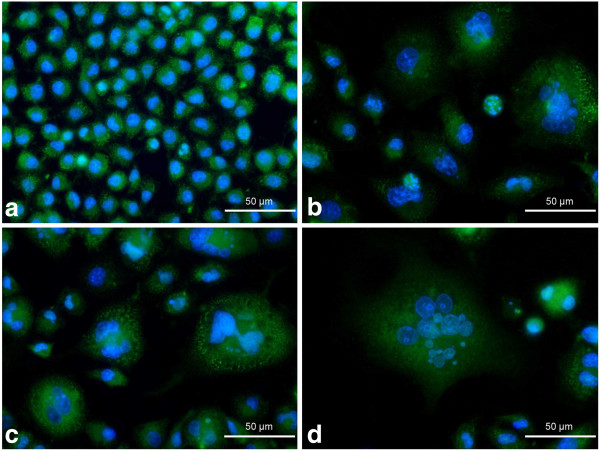
**a-d Immunolocalization of p21**^**Cip1/Waf1/Sdi1 **^**in A549 cells after treatment with etoposide - fluorescence microscopic examination (p21**^**Cip1/Waf1/Sdi1 **^**indirect labeling, secondary antibody was goat anti-mouse IgG-Alexa Fluor 488®). a** Control cells, **b** 0.75 μM etoposide, **c** 1.5 μM etoposide, **d** 3 μM etoposide. Nuclei were counterstained with DAPI. Results are indicative of three independent experiments. *Bar* 50 μm.

**Figure 9:**
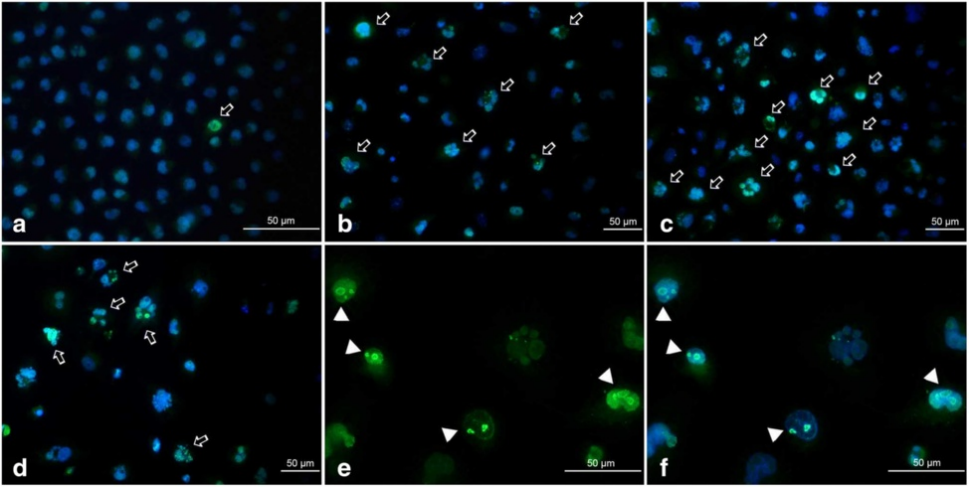
**a-f Immunolocalization of cyclin D1 in A549 cells after treatment with etoposide - fluorescence microscopic examination (cyclin D1 indirect labeling, secondary antibody was goat anti-mouse IgG-Alexa Fluor 488®). a** Control cells, **b** 0.75 μM etoposide, **c** 1.5 μM etoposide, **d**, **e**, **f** 3 μM etoposide. Enhanced fluorescence signal in selected cells and cyclin D1 aggregations indicated (*arrows, arrowheads*). Nuclei were counterstained with DAPI. Results are indicative of three independent experiments. *Bar* 50 μm.

p21^Cip1/Waf1/Sdi1^ immunolocalization showed neither significant induction of expression of this protein nor evident changes in its subcellular localization in the majority of etoposide-exposed A549 cells (Figure 8). As opposed to these results, the alterations of localization and abundance of cyclin D1 were more evident (Figure 9). As a result of the treatment, some cells appeared with an increased overall intensity of intranuclear staining for this protein. Besides that, at the higher concentrations of etoposide (1.5 and 3 μM), the fluorescence signal was often sequestrated in the form of foci, especially in the presumptive nucleolar area (Figure 9e,f).

**Figure 10:**
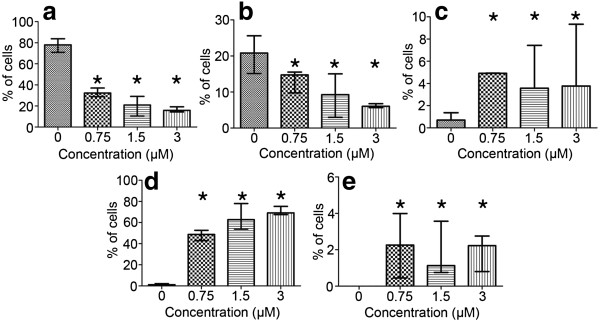
**a-e Progressive changes in the morphology of nuclei in A549 cells after treatment with etoposide - statistical data from microscopic evaluation (staining with DAPI). a** Cells with regular nuclei, **b** cells with nuclei of a regular size and with some morphological abnormalities (lobulation, invaginations, buds), **c** cells with nuclei indicative of apoptosis (apoptotic bodies), **d** cells with large nuclei, **e** cells with features of SAHF formation. Statistically significant differences as compared to the control cells are marked by *asterisks* (Mann–Whitney U statistics, P<0.05). *Columns* - median percentage of cells, *bars* - interquartile range. Results are representative of five independent experiments.

**Figure 11:**
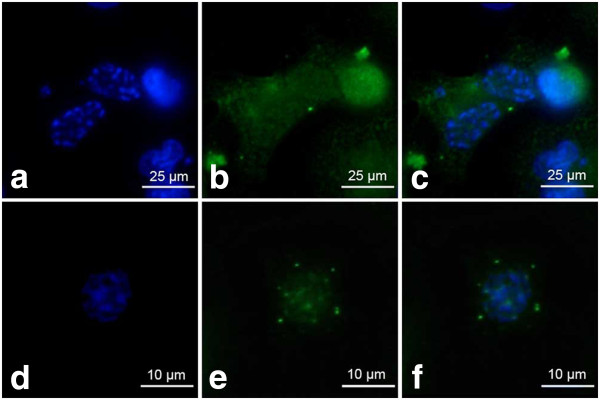
**a-f p21**^**Cip1/Waf1/Sdi1 **^**and cyclin D1 are dispersed in the cells with SAHF – fluorescence microscopic examination (staining as described above). a**-**c** 0.75 μM etoposide, **d-f** 3 μM etoposide; **a**, **d** DAPI, **b** p21^Cip1/Waf1/Sdi1^ labeling, **e** cyclin D1 labeling, **c**, **f** merged. *Upper bar* 25 μm, *lower bar* 10 μm.

Quantification of SAHF and other nuclear abnormalities revealed that the treatment induced profound changes in nuclear morphology, especially the massive appearance of the cells with enlarged nuclei (Figure 10 a-e). Although SAHF formation was induced by etoposide, the percentage of cells presenting these structures was maintained at a stable low level independently of the concentration applied, ranging from 0.45 to 3.99% after the treatment (Figure 10e). Concomitantly, the cells with SAFH manifestation revealed neither p21^Cip1/Waf1/Sdi1^ nor cyclin D1 induction (Figure 11a-f).

### Selected cytoskeletal components - vimentin and G-actin

**Figure 12:**
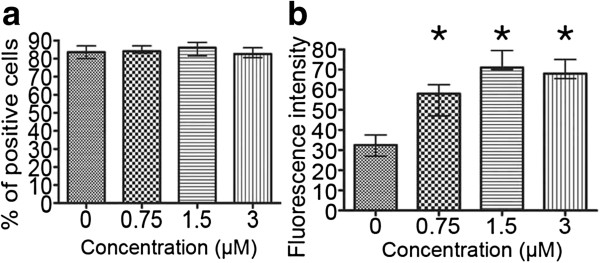
**a-b Quantity of vimentin protein in the A549 population after treatment with etoposide - statistical data from flow cytometric analyses. a** Percentage of vimentin-positive cells, **b** fluorescence intensity reflecting vimentin content. Statistically significant differences were not observed as compared to the control cells (Mann–Whitney U statistics, P<0.05). *Columns* - median percentage of vimentin-positive cells, *bars* - interquartile range. Results are representative of five independent experiments.

Cytometric analysis for the percentage of vimentin-positive cells indicated that there were no statistically significant changes in this parameter resulting from the treatment. Median values ranged between 82-86% for all the concentrations under study and for the control cells, which allowed us to exclude the possibility that some fluctuations in fluorescence intensity might have been caused by an uneven distribution of antibodies (Figure 12a). On the contrary, mean fluorescence intensity, reflecting mean intracellular level of vimentin was increased, which suggests that etoposide acted stimulating on this protein in A549 cells as compared to the control population (Figure 12b). The tendency of these alterations was not homogeneous, as an initial increase at 0.75 and 1.5 μM concentrations was followed by lower, but still statistically significantly high values for 3 μM etoposide.

**Figure 13:**
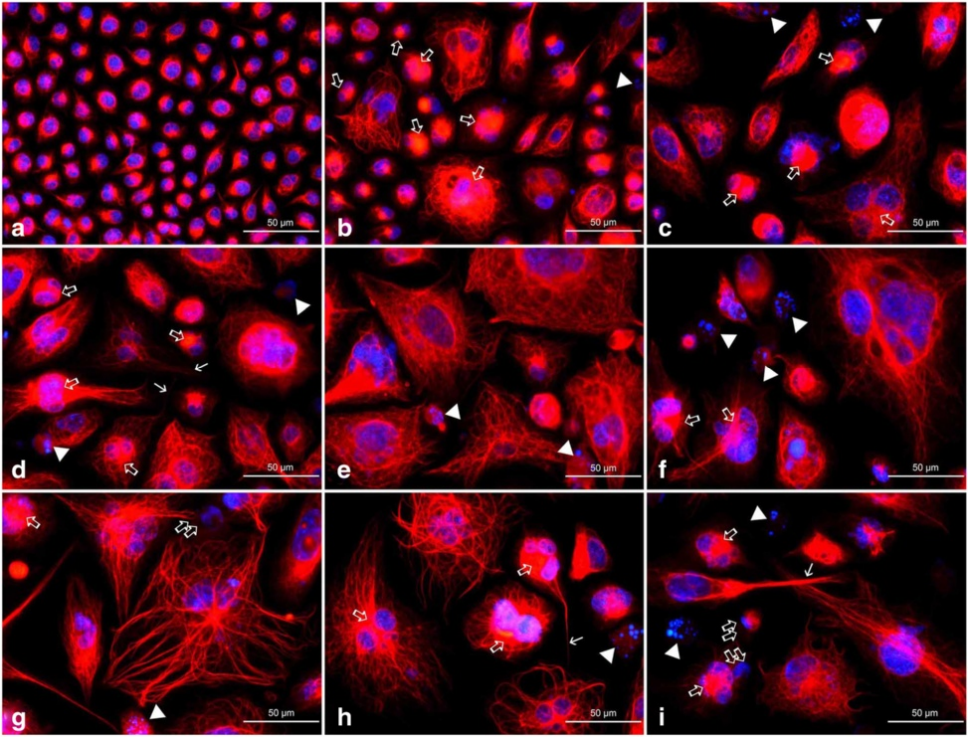
**a-i Organization of vimentin in A549 cells after treatment with etoposide - fluorescence microscopic examination (vimentin indirect labeling, secondary antibody was goat anti-mouse IgG-TRITC). a** Control cells, **b**, **c** 0.75 μM etoposide, **d-f** 1.5 μM etoposide, **g-i** 3 μM etoposide. Apart from regularly organized bundles of filaments in the control cells, abundant, but more heterogeneously organized structures appeared as a result of the treatment. *Thick arrows* point at dense, thick vimentin bundles in the form of septum-like aggregates between sister nuclei, especially common in multinucleated cells, or vimentin threads in the nuclear invaginations; *double thick arrows* - putative budding-like structures in close proximity of the parental cells; *thin arrows* - long vimentin tails; *arrowheads* - diffuse vimentin staining around nuclei with features of *karyorrhexis* or *karyolysis*. Nuclei were counterstained with DAPI. Results are indicative of five independent experiments. *Bar* 50 μm.

**Figure 14:**
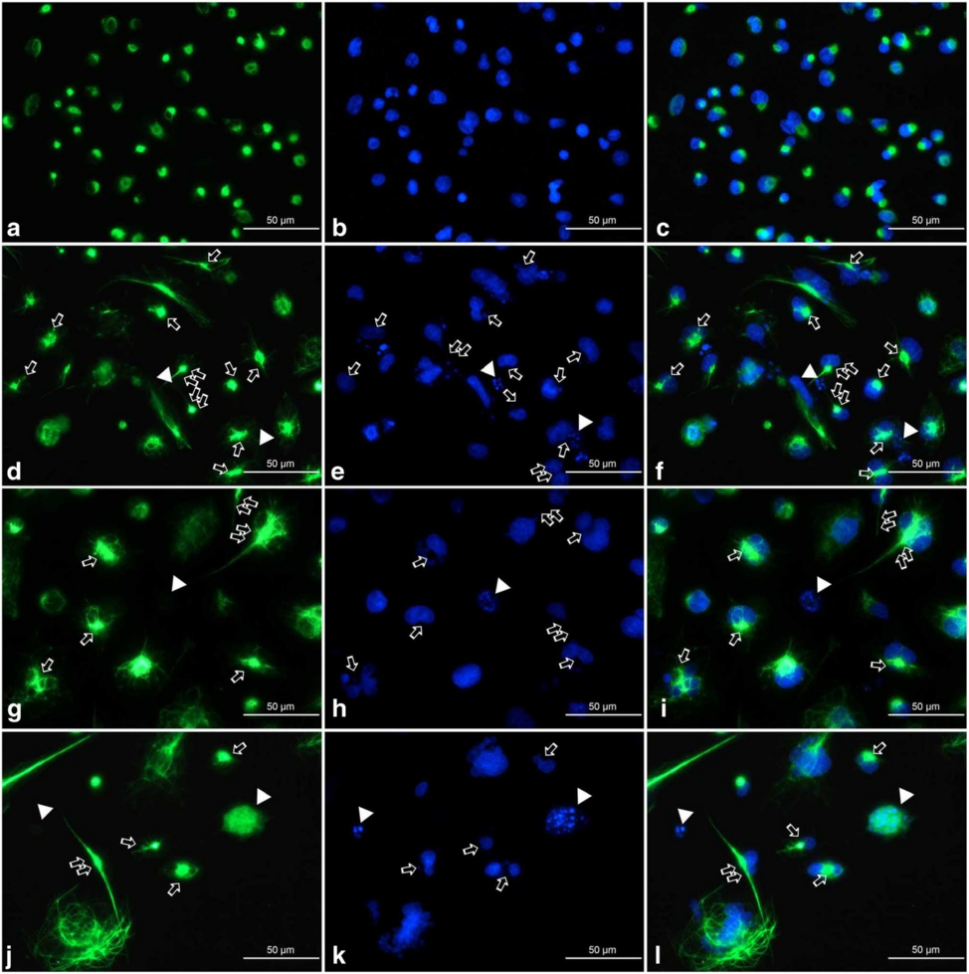
**a-l Organization of vimentin in A549 cells after treatment with etoposide - fluorescence microscopic examination (vimentin indirect labeling, secondary antibody was goat anti-mouse IgG-BODIPY). a-c** Control cells, **d-f** 0.75 μM etoposide, **g-i** 1.5 μM etoposide, **j-l** 3 μM etoposide; **a**, **d**, **g**, **j** vimentin labeling, **b**, **e**, **h**, **k** DAPI, **c**, **f**, **i**, **l** merged. Staining with this method allow us to visualize in a great detail subtle threads of vimentin filaments located in the invaginations of nuclei. Apart from regularly organized bundles of filaments in the control cells, abundant, but more heterogeneously organized structures appeared as a result of the treatment. *Thick arrows* point at thin threads in the nuclear invaginations or more dense vimentin bundles in the form of septum-like aggregates between sister nuclei, especially common in multinucleated cells; *double thick arrows* - cells presenting long vimentin tails (vimentin staining), closely located sister nuclei or thin chromatin threads connecting closely located sister nuclei (DAPI); *arrowheads* - diffuse vimentin staining around nuclei with features of *karyorrhexis* or *karyolysis*. Results are indicative of five independent experiments. *Bar* 50 μm.

**Figure 15:**
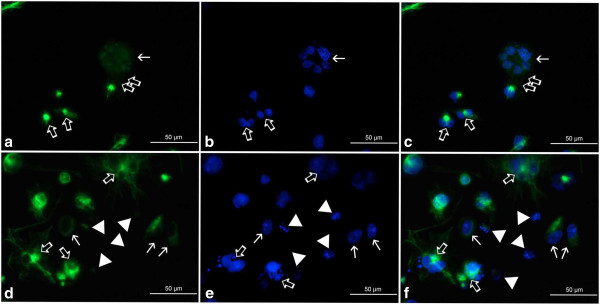
**a-f Organization of vimentin in A549 cells after treatment with etoposide - fluorescence microscopic examination (vimentin indirect labeling, secondary antibody was goat anti-mouse IgG-BODIPY). a-c** 0.75 μM etoposide, **d-f** 3 μM etoposide; **a**, **d** vimentin labeling, **b**, **e** DAPI, **c**, **f** merged. Selected photographs presenting rare nuclei with chromatin structures bearing resemblance to senescence-associated heterochromatin foci (*thin arrows*). These cells featured either disappearing (**a**, **c**) or more regularly woven (**d**, **f**) vimentin scaffold, devoid of threads/foci typical of nuclear invaginations and/or multinucleated cells. *Thick arrows* point at septum-like structures between sister nuclei (**a**, **c**), vimentin aggregates in the perinuclear area of multinucleated cells/cells possessing abnormally-shaped nuclei (**d**, **f**) or nuclei of these cells (**b**, **e**). *Double thick arrows* - putative budding-like structures (**a**, **c**); *arrowheads* - diffuse vimentin staining around nuclei with features of *karyorrhexis* or *karyolysis* (**d**, **f**). Results are indicative of five independent experiments. *Bar* 50 μm.

Fluorescence microscopic observations of vimentin filaments revealed some unique features of their organization after the treatment of A549 cells with etoposide. A well-developed, regular scaffold of subtle, thin and short filaments was characteristic for the untreated control population. In these cells, vimentin filaments formed an evenly distributed cytoplasmic network with most visible foci located in the perinuclear area (Figures 13a and 14a). Etoposide not only induced alterations in the shape and size of cells, but also a heterogeneity in the form and cytoplasmic organization of vimentin intermediate filaments (Figures 13, 14, 15). Starting from the lowest concentration (0.75 μM etoposide), there appeared some enlarged cells presenting thick vimentin fibers or their aggregates in the perinuclear region, along the main axis of the cell, but also more evenly distributed in the cytoplasm (Figures 13b,c, 14d,f, 15a,c). Analogous structures were visible in cells exposed to 1.5 μM etoposide, and featured a fluorescent signal of higher intensity (Figures 13d-f and 14g,i). However, in the well-developed network of vimentin filaments, some less regularly woven spaces appeared, in which organelles and other cytoplasmic inclusions could be located. Populations of multinucleated/large cells seemed to be increased in number. In these, vimentin was abundant and formed ring-like structures, especially around the nucleus (Figures 13, 14 and 15). A faint, dispersed and locally disappearing fluorescent signal was indicative of cells with degenerating cytoskeleton, in which DNA underwent fragmentation or lysis, especially at 3 μM etoposide (Figures 13i, 14j,l and 15d,f). At this concentration, a visible morphological heterogeneity occurred as well. Apart from the large cells with well-developed networks of vimentin filaments, some multinucleated cells presenting intense perinuclear fluorescence were observed, as well as others possessing more dispersed signals that reflect more amorphous vimentin structures and lack of a regular filament scaffold (Figures 13g-i, 14j,l and 15d,f). Most probably, this was related to fragmentation of filaments resulting in the collapse of the cytoskeleton around the nucleus. An interesting fact worth emphasizing is that after exposure of A549 cells to etoposide, vimentin was abundantly present in the invaginations of multi-lobulated nuclei, and especially in the central region of intra-nuclear space between multiple nuclei in multinucleated cells. In addition, we observed only a very few cells featuring chromatin condensations that resemble senescence-like heterochromatin foci. These cells displayed rather subtle and regularly-woven filament scaffolds, devoid of the presence of similar aggregating structures in the perinuclear space (Figure 15).

**Figure 16:**
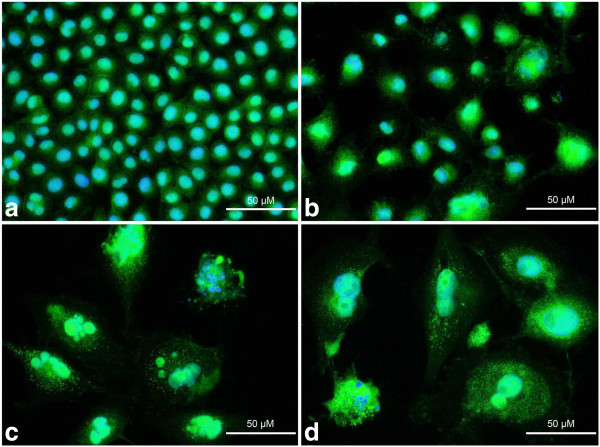
**a-d Localization of G-actin in A549 cells after treatment with etoposide - fluorescence microscopic examination (labeling with DNase I). a** Control cells, **b** 0.75 μM etoposide, **c** 1.5 μM etoposide, **d** 3 μM etoposide. Visible aggregation of G-actin in the perinuclear area after the treatment, as well as an increased number of G-actin-positive dot-like and vesicle-like structures in the cytoplasm. The intense fluorescence patterns indicative of cells with DNA fragmentation. Nuclei were counterstained with DAPI. Results are indicative of five independent experiments. *Bar* 50 μm.

**Figure 17:**
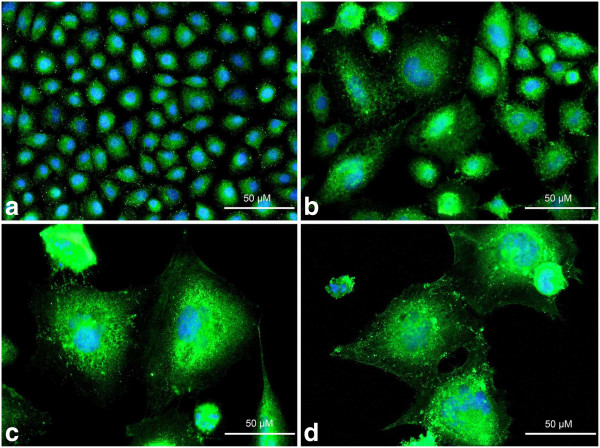
**a-d Localization of G-actin in A549 cells after treatment with etoposide - fluorescence microscopic examination (labeling with VDBP). a** Control cells, **b** 0.75 μM etoposide, **c** 1.5 μM etoposide, **d** 3 μM etoposide. Visible aggregation of G-actin in the perinuclear area after the treatment, as well as in the form of dot-like structures in the cytoplasm. The intense fluorescence patterns indicative of cells with DNA fragmentation. Nuclei were counterstained with DAPI. Results are indicative of five independent experiments. *Bar* 50 μm.

Both methods employed for G-actin analysis (i.e. with DNase I and VDBP) suggested a rather uniform localization of this component in the control A549 cells in comparison with the etoposide-exposed population, in which more intense staining was characteristic of the perinucear region of the cell (Figures 16 and 17). These alterations were especially evident in large cells possessing segmented nuclei or in multinucleated cells resulting from the treatment (Figures 16 b-d and 17 b-d). The perinuclear foci were similar to ring-like vimentin structures, but less regularly organized. Some aggregates, most probably reflecting the process of actin filament nucleation, were found in the central and cortical parts of the cytoplasm. Other structures detected via DNase I labeling, suggest either an increased number of G-actin-positive vesicles, which may have been formed during extensive exocytosis/DNA degradation in response to etoposide, or may simply result from DNase I affinity to some mitochondrial components, in which case they reflect oxidative-stress induced formation of these organelles (Figure 16). Nevertheless, most intense fluorescence was observed in the cells presenting a cell death phenotype defined by the presence of pyknotic or fragmented nuclei, irrespective of the staining method employed (Figures 16c,d and 17c,d).

A relative G-actin content in the cell nucleus was estimated after treatment with etoposide as compared to the untreated cell population. Statistically significant differences were not observed, only some minor fluctuations in fluorescence intensity of G-actin appeared as a result of the treatment. G-actin fluorescence intensity in negative control cells was also stable (data not shown). These indications were further supported by preliminary confocal microscopic examination.

## Discussion

Senescence on the cellular level is believed to be a program of irreversible cell cycle arrest, in which the cell stays unresponsive to mitogenic stimulation. However, metabolic activity of the cell is retained, resulting in the appearance of some morphological manifestations that may be defined as a senescent cell phenotype, and include enlargement accompanied by flattening of the cell, enhanced activity of senescence-associated ß-galactosidase, lipofuscin accumulation, as well as increased cytoplasmic granularity and nuclear volume. Some further alterations related to the nucleus and its functions comprise formation of SAHF and, above all, changes in the expression pattern of tumor-suppressor genes, e.g. *p53*, *p21*^*Cip1/Waf1/Sdi1*^, *p14*^*Arf*^, *p16*^*Ink4a*^, *pRb*[16-19].

However, on the basis of the results obtained in the present study, as well as our previous report [20] and insight gained from experiments of others, we suggest that there should be a clear distinction between senescence on the cellular level and senescence of the whole population of cells, e.g. cell line models. The first observations regarding these phenomena came from the studies of Hayflick, who presented a restricted proliferative capacity of cellular populations *in vitro*[21]. Later, analogical characteristics were shown to exist in cancer cell lines as a result of chemotherapeutic treatment and other therapeutic modalities [22-24], although these cells were previously regarded to be "immortal" due to unrestrained proliferation and telomerase expression. Moreover, recently the clinical impact of the senescence program, as well as the pros and cons of its induction have been broadly discussed [25-28]. At the same time, the lack of a clear definition and unequivocal markers indicative of this phenomenon, makes it particularly difficult to firmly establish its contribution to the final outcome of therapy. All the more so because some features are misleadingly perceived as crucial determinants of the program. These features could include enhanced senescence-associated ß-galactosidase activity and polyploidy induction.

As regards polyploidy induction, it has been documented that cell population undergoing replicative senescence may feature a defective mitotic checkpoint, which eventually leads to aberrant mitoses [29]. In view of these findings, it cannot be ruled out that some nuclear alterations accompanying polyploidy reflect a crisis-like event, including some breakage-fusion-bridge cycles that result in increased genomic instability. In our research, a G_2_/M population was increasing in parallel with polyploidy induction, which may rather suggest an endoreplication-like response after mitotic slippage. At the same time, the extent of G_2_/M accumulating cells as well as SAHF containing cells seemed to be relatively low as compared to those manifesting enhanced SA ß-galactosidase, a fact which may, along with polyploidization, suggest an unstable senescence-like program induced by etoposide. Also results of the TUNEL method imply an accumulation of cells from G_0_/G_1_ and S phases of the cell cycle into G_2_/M and transient arrest followed by some endoreplication/cell death events. Due to the fact that extensive DNA fragmentation accompanied these alterations, chromosomal instability and breakage-fusion-bridge cycles could not be excluded. Similar results were obtained by Sliwinska et al. (2009) after treatment of HCT116 cells with doxorubicin, another topoisomerase II inhibitor. The authors claimed that a substantial fraction of the cells underwent endocycling without entering mitosis, and that some dividing polyploid cells produced aneuploid progeny [30].

DNA damage signaling has been proved to be coupled to its replication in direct response to topoisomerase inhibitors, including etoposide in the A549 cell population. On the one hand it suggests the induction of DSB following collision of cleavable complexes with replication forks, but some DSB also appeared in non-replicating cells, which may be indicative of other mechanisms of action of this drug, i.e. oxidative stress generation or collision of cleavable complexes with transcription machinery [31]. On the other hand, it is tempting to consider its influence on mitotic cells and the involvement of the DNA replication process in the long-term response to etoposide. Our results obtained from the TUNEL method confirm the findings of Zhao et al. (2012) regarding the lack of cell-cycle specific effect on DSB induction. However, in our study, G_2_/M and polyploid fractions apparently accumulated the highest numbers of damaged cells, which may imply some abnormal/abortive mitoses accompanied by endoreplication-like events. This interpretation is further supported by the fact that in our investigation, regular mitotic figures were only observed exceptionally in etoposide-exposed cells, whereas nuclei presenting atypical morphology were rather abundant. In addition, the TUNEL-positive cells were more abundant after treatment in comparison with the cells presenting biochemical features of cell death in the annexin V/PI assay. There is clear evidence that endo-polyploidization in senescent cell population constitutes a consequence of re-replication of genomically-damaged G_2_/M cells [32]. At the same time, independent karyoplasts/reproductive genome-reduced subcells may form from giant polyploid cells via some kind of reductional division, resulting in increased genetic variation [33,34]. What is more, induction of DNA damage in cancer cell lines was proven to be related not only to endopolyploidy and depolyploidization, but also to overexpression of meiosis-specific genes and mobilization of their products to the sites strategic for division [35-37]. More recently it has also been reported that although there are some morphological and biochemical features of senescence as well as growth arrest in the population of A549 cells exposed to topoisomerase II inhibitors [20,38], long term effects of the treatment include a re-growth of a fraction of population originating most probably from cancer stem cells [38]. In this situation, a somewhat more prolonged, but still reversible growth arrest of the population occurred after inhibition of the DNA damage response pathway. Due to the fact that 24 h after exposure to etoposide we still observed a relatively high number of TUNEL-positive cells in comparison with those annexin V/PI-positive, this pathway was most probably active in our experiment as well.

Hence, some alterations in heterochromatin features, like gluey or stick heterochromatin in near-senescent population of cultured cells, seem to be responsible for mitotic failure and chromosomal instability [39]. Analogically, etoposide may induce impairments in untangling of chromatin, which in turn interferes with the proper formation of chromosomes and the decatenation of chromatids [5], leading most probably to the same final outcome, i.e. induction of a senescent-like phenotype in the cancer cell population along with increased polyploidy and genetic instability, as well as heterogeneity of the response. What is more, an unstable senescence program induced in cancer cell populations by DNA damage is thought to be related to a phenomenon corresponding to the above-described series of events, and termed neosis [40,41]. In fact this process may contribute to chemotherapy resistance, due to the fact that increased instability along with repeated cycles of self-renewal may favour the accumulation of survival-promoting mutations and the expansion of more invasive subclones, especially under continuous contact with a chemotherapeutic drug [37]. Moreover, polyploidy was suggested to be a transiently appearing response to treatment, followed by differentiation of the genetic material into subnuclei that are either targeted to survival or to autophagic degradation [42].

As regards the second above-mentioned senescence marker, i.e. senescence-associated ß-galactosidase, the activity of this enzyme reflects changes in lysosomal content and functions, and consequently the altered cellular metabolism of aging cells [16,43-45], which does not interchangeably mean a cell cycle-arrested cell. This is also supported by our observations of the cell ultrastructure following the treatment, which revealed abundantly present large lysosomes containing numerous inclusions of degradable and non-degradable materials such as lipofuscin, but also some organelles or their components. However, as an increased lysosomal mass may also appear in other processes such as, for example, autophagy, as evidenced by acridine orange staining [46], an enhanced activity of this enzyme may be indicative of this kind of metabolic shift as well. Therefore, senescence-associated ß-galactosidase activity should not be regarded as the sole marker of the senescence program on the cellular level. Moreover, our results showed an uncoupling between an enhanced activity of this enzyme and cell cycle arrest, as the tendencies of these two parameters seemed not fully consistent. Analogical results were obtained by Dulić et al. (2000), who reported a discrepancy between the senescence-like phenotype and cell cycle block in human diploid fibroblasts transfected with HPV *E6* oncogene that contributed to p53 degradation and in *p21*^*Cip1/Waf1/Sdi1*^ nullizygous mouse embryonic fibroblasts [47]. Thus, being neither a crucial determinant nor an unequivocal reflection of stable cell cycle arrest, an enhanced senescence-associated ß-galactosidase activity rather reflects a metabolic response of the cell to stress. Nevertheless, it has been recently reported that autophagy mediates a transition of mitotic cells to senescence, especially influencing the phenotype, and that autophagic degradation of DNA is an important factor during segregation of subnuclei in polyploid cells [42,48]. These suggestions are also consistent with the particularly high activity of this enzyme in polyploid A549 cells resulting from the treatment with etoposide observed in this study. What is more, in our fluorescent microscopic, as well as transmission electron microscopic analyses, we were able to distinguish some diffuse DAPI staining, DNase I staining and some dispersed chromatin-like structures in the cytoplasm of A549 cells after treatment. This supports the hypothesis of DNA degradation. It therefore seems that an increase in the activity of senescence-associated ß-galactosidase is particularly related to phenotypic alterations in the senescent cell population. Morphological changes typical of a senescence-like response were also confirmed by our observations at the ultrastructural level, which was evident from increased heterogeneity of the cytoplasmic compartment, but also progressive alterations of the nuclei. The latter were most probably a phenotypic manifestation of abnormal mitoses/mitotic catastrophe.

Some hallmarks of the senescence phenomenon strictly related to the execution of a cell cycle arrest are believed to be changes in expression of the cell cycle regulatory molecules, including cyclin-dependent kinase inhibitors, cyclin-dependent kinases and cyclins, as well as formation of SAHF [18,23,25,26,30]. p21^Cip1/Waf1/Sdi1^ was previously reported to be a reliable marker of prematurely induced senescence in many cancer cell lines. Particularly, profound alterations in the expression of the gene encoding this protein and/or in the localization of its product were observed, which may be the major contributor to the G_2_/M arrest, but also may activate the G_1_ checkpoint [30,49]. For example, Sliwinska et al. (2009) found lack of Ki-67 expression along with increased expression of p21^Cip1/Waf1/Sdi1^ and cyclin D1 in HCT116 cells after incubation with a low dose of doxorubicin. As regards their fluorescence microscopic observations, the large senescent-like cells manifested evident bright signals for p21^Cip1/Waf1/Sdi1^[30]. To refer more specifically to the A549 population, we would like to mention the work of Shen and Maki (2010). The authors documented there that p21^Cip1/Waf1/Sdi1^ induction and its nuclear accumulation were indispensable for maintenance of a senescence-associated G_1_ cell cycle arrest of A549 cells with unreduced DNA content (4N) after transient Nutilin-3a treatment, with knock-down of *p21*^*Cip1/Waf1/Sdi1*^ or *p53* resulting in endoreduplication [49]. These findings are particularly interesting in light of our observations, which did not reveal significant changes in p21^Cip1/Waf1/Sdi1^, supporting the hypothesis of an unstable senscence-like state induction that was accompanied by endocycling events in our conditions. As reported earlier, insufficient p21^Cip1/Waf1/Sdi1^ activity in human fibroblasts with morphological features of a senescence program may lead to uncoupling between phenotypic features of the senescence program and cell cycle arrest [47], in accordance with our results as well. Further evidences also reinforce this point of view, indicating that in the absence of the proper p21^Cip1/Waf1/Sdi1^ function, cells may undergo a transient G2-like arrest, which is followed by replication without regular intervening mitoses [50].

We think that such a compromised senescence phenotype or senescence-like state may be a consequence of the mutations identified in A549 cells, in particular homozygous deletion of the *Ink4b/Arf/Ink4a* locus [8-11]. This, in turn, may render the cells incapable of activation of not only p16^Ink4a^, but also p14^Arf^, which is an upstream regulator of p53-mediated p21^Cip1/Waf1/Sdi1^ induction via HDM2 sequestration. Previous works aimed at elucidation of the functions that are specifically fulfilled by p16^Ink4a^ and p21^Cip1/Waf1/Sdi1^ during senescence execution and suggested their complementation for the most efficient induction and maintenance of the program [51]. The circumstances vital for the irreversibility of senescence are presently being determined for both the Rb and p53-mediated pathways, in which the above-mentioned proteins constitute essential players. These involve the positive feedback loop of ROS accumulation in case of p21^Cip1/Waf1/Sdi1^ and SAHF formation in case of p16^Ink4a^ -Rb [52,53]. Therefore, in order to verify a potential contribution of the p16^Ink4a^ -Rb arm that may result in the G_0_/G_1_ arrest, we checked the status of cyclin D1 and SAHF formation in our study.

In agreement with other reports describing accumulation of cyclin D1 in senescent cells and our results with the HL-60 cell line (manuscript in preparation), here we showed some changes in the amount and localization of this protein in a senescence-like state. Increased cyclin D1 level is regarded by some authors as a feature of senescence-like conditions that are accompanied by polyploidization events [30,54-56]. At the same time, the major role of this protein is to act as an antagonist of Rb-mediated cell cycle arrest and to drive the cell cycle progression through G1 phase [57]. Therefore, it should not be surprising that an enhanced expression of this protein may be followed by an unfavorable prognosis, especially if it is activated during senescence-like conditions [30,56]. Many reports concerning cyclin D1 in senescence, refer in fact to a senescence-like state in the population of heterogeneous by nature cancer cells. The recent report of McGowan et al. (2012) clearly demonstrated the possible latent effects of nuclear accumulation of cyclin D1 in a population of MCF-7 cells presenting typical senescence-like morphology as a consequence of the 14^Arf^-p53-p21-Rb pathway activation. These effects were predominantly related to relocalization of Ki-67 into the nucleoli, increased genomic instability and abnormal cell divisions [56]. Such morphological features and nuclear cyclin D1 accumulation were evident also in our experiment, suggesting, along with low frequency of SAHF, the instability of the induced state. As regards nucleolar sequestration of the cyclin D1 protein observed by us, the phenomenon may have several alternative implications for senescence execution, which presently remain obscure. First of all, sequestration of other cell-cycle regulators contributes to their inactivation [58]. Bearing in mind the morphology of the cells that accumulated cyclin D1 and only faint, dispersed staining in the cells with SAHF, it may also be hypothesized that sequestration probably serves the special purpose of inactivation of the protein on a cellular route to senescence. However, the phenomenon requires a more in-depth revision, especially in face of the fact that mammalian cyclin D1/Cdk4 complexes have been proven to contribute to endoreduplication, most probably via the stimulation of nucleoli enlargement and ribosomal biogenesis [59]. In our study the nuclei of polyploid cells showed often intense cyclin D1 staining, sometimes accompanied by intranucleolar aggregations as well.

In this situation, if the cyclin D1 sequestration had been involved in the execution of a cell cycle arrest, we would have been able to observe an increased number of the cells with SAHF manifestation. Derepression of Rb after inactivation of cyclin D1, may have led to supression of the E2F target genes crucial for replication and, finally, to activation of the SAHF pathways essential for irreversibility of the program. In fact, this was not the case in our conditions. Instead, we found the cells with widespread DNA damage and abnormal morphological features indicative of inappropriate DNA replication and aberrant proliferation.

In order to gain a more in-depth insight into the state of A549 cells following exposure to etoposide, we referred also to analyses of some previously described cytoskeletal indicators of senescence, i.e. G-actin and vimentin.

Vimentin is mainly expressed in cells of mesenchymal origin and has been described as a marker of epithelial-to-mesenchymal transition, which is a phenomenon related to increased invasiveness and resistance of some cancer cells, thus manifesting the progression of cancer [60,61]. On the other hand, there are studies documenting an abundant vimentin presence, as well as its special role in phenotypic changes that accompany senescence phase in cell populations [12,15,62].

Here we would like to suggest for the first time a special role for vimentin in multinucleated dividing cells resulting from etoposide exposure. Since the protein accumulates especially in the intranuclear spaces of multinucleated cells which are expected to give origin to aneuploid progeny, as well as in invaginations of lobulated nuclei, it is highly probable that it also contributes somehow to the formation, separation, segregation and isolation of nuclei with reduced DNA content, which, according to previous reports [33,42], is followed by the budding of individual karyoplasts/paradiploid descendants from the parental nucleus. Darkly stained structures were observed by Walen (2005) in the division furrow during amitotic divisions of polyploid nuclei in a senescent cell population [33]. Our morphological observations strongly suggest the involvement of vimentin in this process, because the most intense fluorescence signal was also evident in division axis between putative daughter nuclei identified by their aberrant morphology, different sizes and shapes. In these cells, similar to the study of Walen (2005), we noticed the presence of some division-like furrows starting from invaginations that were in close proximity to vimentin-rich areas of the cytoplasm, as well as small distances between sister nuclei, filled with septum-like vimentin structures, which suggests amitotic divisions. Cytoplasmic tails, most probably indicative of the process of karyoplast budding, were also present in both studies, and in our case they were shown to be vimentin-rich. Additionally, in our experiment aggregation of this protein was particularly evident around multiple nuclei of giant cells, often at the expense of its distribution in the perpiheral cytoplasmic areas, which on the whole resembled the process of vimentin retraction into the form of ring-like structures. It has previously been shown that vimentin participates in cytokinesis, being expressed in a cleavage-furrow specific manner. Its mutant form, unresponsive to Aurora B phosphorylation, left daughter cells unseparated [63]. In light of these findings and our results it seems especially interesting that giant cancer cells appearing after irradiation and expressing catalytically active Aurora B kinase, also retain the capacity to divide [64]. Some multinucleated cells presented by these authors also had features of abnormal divisions including uneven distribution of chromatin into daughter nuclei, the presence of thin chromatin threads and cytoplasmic tails connecting dividing cells. Besides that, previous studies have indicated that intermediate filaments are crucial for regulation of germ cell nuclear behaviour, participating in meiosis, selection of meiotic products, formation of the gametic pronucleus, its transfer across cell-cell junctions and, finally, in pronuclear fusion during zygote formation in *Tetrahymena*[65]. Intermediate filament-related protein MNS1 is also thought to contribute to the organization of nuclear morphology during meiotic prophase [66]. In addition, the activity of the isolated head domain of vimentin, released by HIV-1 protease, has been shown to be both necessary and sufficient for alterations of nuclear shape including the formation of invaginations and extensive lobulation, as well as for chromatin reorganization [67].

In further agreement with our hypothesis, rare cells presenting heterochromatin morphology indicative of SAHF, which may imply more stable cell cycle arrest, presented quite the opposite vimentin organization, i.e. rather regular, faintly-woven, occasionally even dispersed structures. Thus far there have been contradictory indications related to senescence-associated vimentin regulation, as well as its possible function in the program. Firstly, overproduction of vimentin is considered to be indicative of senescence phase in cultured cell population [12], however, depending on the tissue or cell type, its expression may also be diminished [68]. Secondly, apart from its anti-oncogenic and suppressive functions during cellular transformation, the possible involvement of this protein in senescence delay and spontaneous transformation has been proposed [12,69]. Vimentin is believed to contribute to recombinational repair of nuclear and mitochondrial DNA [69] and, in line with these observations, this cytoskeletal protein may participate in cytoplasmic anchorage of p53 during senescence [15], thus making it unavailable for its transcriptional targets and, as a consequence, limiting its nuclear-dependent tumor suppressor functions and downstream pathways of cell death and/or cell cycle arrest. However, as opposed to senescence, which is considered to be a tumor-suppressive mechanism, other reports also suggest an involvement of this protein in immortalization and epithelial-to-mesenchymal transition, both events related to carcinogenesis and tumor progression. However this may only be an apparent contradiction, because a senescent cell population constitutes a source of extended life cells, participating in transformation to immortalized ones [33]. What is more, since it is engaged in extensive repair processes, this protein has the potential to promote increased plasticity of the genome and invasiveness of cancer cells [69]. Interestingly, further evidence of vimentin’s contribution to the integrity and repair of the genome is the fact that the destruction of this cytoskeletal component during early stages of apoptosis actually reinforces a pro-apoptotic signaling cascade in the cell, via the product of its proteolysis [70-72], thus making it an attractive candidate among possible „point of no return” sensors. Here we would like to point out the difference in vimentin organization between cells with typical manifestations of SAHF, the multinucleated cells containing abnormally-shaped, irregular nuclei including micronuclei, their putative descendants, as well as the apoptotic cells. Additionally, our cytometric analyses support indications of an increased vimentin content in the senescent cell population as a whole, which may reflect not only a shift in morphology towards an increased cell volume, but also accumulation of vimentin in the cytoplasmic areas crucial for the separation of individual nuclei.

G-actin has previously been described as a marker of senescence, and further studies revealed that especially its nuclear accumulation may be correlated with cell cycle arrest in G_1_[13,73]. Nuclear G-actin pool was visibly increased not only in replicative, but also in stress-induced premature senescence of human diploid fibroblasts, which was also accompanied by alterations in the expression and distribution of phosphorylated Erk1/2 kinases and small G proteins (belonging to the Rho family). These changes have been interpreted as a reflection of impairments in nuclear export of actin resulting from improper regulation of MEK signaling pathway [73]. In turn, subsequent observations have shown that the increase in G-actin pool may also be related to the presence of cofilin in the nuclei of senescent G_1_-arrested fibroblasts, because cofilin appeared there in its active, dephosphorylated form, capable of F-actin fragmentation [13]. In support of this, it was also reported that dephosphorylated cofilin-dependent nuclear import of actin occurs in response to stress [74] and that analogical stress-induced events are regulated independently of apoptosis [75]. Moreover, there are some indications of possible interrelations between nuclear actin and proliferative capacities of the cell. These suggest that an enforced G-actin export from the nucleus induced not only the reversal of phenotypic and biochemical senescence features, but also stimulated cell cycle progression and replication of previously G_1_-arrested cells [13]. On the other hand, impairments in G-actin export were related to growth arrest and the appearance of senescent morphology [13,76]. Furthermore, nuclear G-actin accumulation is considered to be an early and universal marker of senescence in the cell, including senescent cancer cells [13].

In our study, microscopic quantitative analyses of G-actin content in the nucleus area did not show significant changes after etoposide exposure. However, we believe that this is consistent with previous reports suggesting that dephosphorylated cofilin is a crucial factor for nuclear G-actin accumulation and stable G_1_ cell cycle arrest. In fact, we observed an increase only in G_2_/M population, in which cofilin levels are supposed to be the lowest. Thus, the lack of nuclear G-actin accumulation in this study further reinforces our assumption that unreduced G_2_/M cells were not irreversibly arrested in G_1_, but rather underwent endoreplication. However, some cytoplasmic G-actin alterations were evident and we suggest they may be caused by several synergistically acting events, including oxidative stress generation, cell death induction, exocytosis and changes in the cell morphology. Oxidative stress is known to influence the amino acid sequence of actin, via modifications in residues critical for the process of polymerization [77]. In accordance with this, and with previous studies documenting dose-dependent depolymerization of F-actin under the influence of etoposide [78], we observed here a more prominent cytoplasmic staining for G-actin with both methods employed. Based only on DNase I labeling, we would not have been able to exclude the possibility that this more intense signal had in fact indicated an enhanced mitochondrial biogenesis and/or autophagic DNA degradation in response to etoposide, as has previously been suggested [42,79].

The most intense fluorescence of G-actin was documented in this study for cells presenting apoptotic-like morphology, including nuclear fragmentation and the formation of apoptotic bodies, which was most probably also indicative of F-actin cytoskeleton destruction, but may also imply an involvement of actin in the development of morphological symptoms and/or cell death execution. For example, it has been shown that actin filaments aggregate at sites of apoptotic bodies formation [80]. Apart from that, early stages of apoptosis are characterized by low expression of actin, subsequently followed by F-actin reorganization and accumulation of G-actin in apoptotic bodies [81]. In light of these findings, it also seems meaningful that G-actin functions as an inhibitor of DNase I [82-84] until the proteolytic cleavage in advanced stages of apoptosis. Furthermore, inhibition of the proteolytic cleavage of actin results in the suppression of DNA fragmentation in etoposide-treated cells, and mutation of the cleavage-site may actually lead to cellular transformation [82,85]. The presence of actin was shown to be crucial for transduction of necrosis-related signaling into mitochondria [86].

## Conclusions

In conclusion, although the occurrence of a stable senescence program in single etoposide-exposed cells could not be ruled out, e.g. as a result of mitotic block in cells with failed cytokinesis [87], a great deal of caution should be advised when interpreting a general response to etoposide in A549 cell population as a senescence phenomenon. In this study, SAHF, which are believed to contribute to cell-cycle exit via suppression of proliferation-driven genes, were observed only exceptionally. Moreover, although G_2_/M arrest occurred, it was accompanied by a concomitantly increasing fraction of polyploid TUNEL-positive cells, which, along with the lack of nuclear G-actin accumulation seems to exclude a stable G_0_/G_1_ arrest of cells with unreduced DNA content. We suggest that a delicate balance between self-renewal and senescence in A549 cells may be affected as a result of impairments in two important senescence pathways, i.e. via p53 and pRb. This, in turn, may be a consequence of the genetic background of A549 cell line, i.e. homozygous deletion of the *Ink4b/Arf/Ink4a* locus. In agreement with these presumptions, it has been previously documented that *p53* and/or *p16*^*Ink4a*^ defects may contribute to the proneness of some lung cancer cells to senescence escape [27]. In our microscopic observations we were also unable to show significant induction of either p21^Waf1/Cip1/Sdi1^ or prominent SAFH formation, supporting the suggestion that some features of senescence may appear in this situation independently of a cell cycle arrest and/or secondarily, as a consequence of abnormal mitosis/polyploidization. Aberrant regulation of cyclin D1 may also contribute to this phenotype, which requires further studies.

Thus, the response of the A549 population to etoposide may be described as heterogeneous, demonstrating not only some features of cell death, but also broad polyploidization events typical of a pre-senescent stage, as well as the senescence-like phenotype manifesting itself mainly as a kind of metabolic shift, resembling autophagic demolition of the cellular content. This seems particularly interesting in light of recent findings suggesting the involvement of autophagy in the development of morphological symptoms at a pre-senescence stage [42,48,88,89], but the precise role of this program is yet to be determined. Besides that, pre-senescent tetraploid/polyploid cells were shown to coordinate DNA-damage response, self-renewal and senescence signalling pathways [42,90,91], constituting a potential source of extended-life cells or, in cancer cell populations, cancer stem cells [37,38]. Apart from that, the morphological senescence-like alterations observed by us were unaccompanied by a stable cell cycle arrest.

Last but not least, here we present preliminary morphological evidence for the possible role of vimentin in depolyploidization of giant cancer cells appearing as a result of a pre-senescent stage induction.

## Methods

### Cell culture

A549 cells were grown in monolayer in Dulbecco's modified Eagle's medium with Glutamax (DMEM; Gibco) with 50 μg/ml gentamycine (Sigma-Aldrich) and supplemented with fetal bovine serum (FBS; Gibco) at a final concentration of 10%. Cell cultures were maintained at 37°C in a humidified CO_2_ incubator.

Etoposide (Sintopozid®) was obtained from S.C. SINDAN S.R.L., Bucharest, Romania. The drug was kept in accordance with the supplier's recommendations, and working solutions were prepared in fresh medium prior to use. Cells in the exponential phase of growth, 24 h after seeding, were incubated with increasing concentrations of etoposide (0; 0.75; 1.5; 3 μM) for 72 h. Before further experimental procedures, the cells were cultured for an additional 24 h in a fresh drug-free medium.

### Senescence-associated ß-galactosidase assay

Senescence-associated ß-galactosidase activity was determined using the Senescent Cells Staining Kit (Sigma-Aldrich), in accordance with the enclosed instructions. After rinsing with Dulbecco's PBS, the cells were fixed on coverslips with a solution containing 2% formaldehyde/0,2% glutaraldehyde for 6 min at room temperature. After being washed in DPBS, the cells were stained with a freshly prepared solution (pH 6) including X-gal (5-bromo-4-chloro-3-indolyl-ß-galactopyranoside) in the dark at 37°C for 20 h. After that, the coverslips were rinsed again and mounted with Aqua Poly/Mount (Polysciences).

The percentage of cells displaying a visible reaction in the presence of indigogenic substrate was established microscopically (Eclipse E800, Nikon), and was presented as the mean value from five fields for each experimental condition, from at least ten independent repeats.

### Cell cycle analysis

Cells growing on 6-well plates, after trypsinization and washing, were suspended in PBS (1–2 × 10^6^ cells/ml). The cell pellet obtained from centrifugation of suspensions (5 min, 300 × g) was resuspended in 1 ml of NSS solution containing 50 μg/ml PI, 0.01% (w/v) sodium citrate (Sigma-Aldrich) and 0.03% (v/v) nonylphenylpolyethylene glycol (Nonidet P40 Substitute; Fluka). Following centrifugation (5 min, 300 × g), the cells were resuspended in 250 μl of NSS and stained at room temperature in the dark for 15 min. Finally, each sample was incubated with 250 μl of RNase A solution (at a concentration of 10 μg/ml in PBS) (Sigma-Aldrich) for 15 min at room temperature in the dark. After the addition of 0.5 ml of PBS, flow cytometric analysis was performed on a FACScan flow cytometer (Becton-Dickinson). Cell cycle distribution was estimated for 20 000 events using CellQuest software (Becton-Dickinson). The whole cell population was divided into fractions with respective DNA contents (G_0_/G_1_; S; G_2_/M; <G_1_; >G_2_).

### TUNEL assay

A commercially available kit (APO-DIRECT, BD Biosciences Pharmingen) was used to perform the terminal deoxynucleotidyl transferase-mediated dUTP nick-end labeling (TUNEL) method analysis to assess DNA fragmentation rate. Following trypsinization, cells were fixed with 1% (w/v) formaldehyde (Polysciences) in PBS, 15 min, on ice and then washed with PBS. Before staining, the cells were resuspended in 70% (v/v) ice-cold ethanol (POCh) and kept at -20°C for at least 18 h. After fixation and permeabilization, the cells were incubated with staining mixture containing TdT enzyme and substrate (dUTP-FITC) at 37°C. Counterstaining was performed with PI/RNase solution. Finally, the cells were analyzed on FACScan using CellQuest software (Becton-Dickinson). 20 000 events were acquired and non-clumped cells were gated.

### Annexin V assay

The extent of apoptotic and necrotic cell death was evaluated based on double staining with annexin V-FITC and PI using the Annexin V-FITC Apoptosis Detection Kit (BD Biosciences Pharmingen). The procedure was performed according to the enclosed protocol. Briefly, after trypsinization and washing of the cells with PBS, they were resuspended in annexin V binding buffer including 2.5% annexin V-FITC (v/v) for a 15-min incubation (dark, RT). After centrifugation (5 min, 300 × g), annexin V binding buffer with 5% addition of PI was incubated with the resulting pellet for 5 min in the above-mentioned conditions. Finally, flow cytometric analysis (FACScan, CellQuest software; Becton-Dickinson) allowed us to determine the percentages of cells presenting biochemical features of early apoptosis (annexin V-positive/PI-negative), late apoptosis (annexin V-positive/PI-positive), necrosis (annexin V-negative/PI-positive), as well as viable cells (annexin V-negative/PI-negative).

### Immunofluorescence

#### Vimentin labeling

Cells grown on coverslips in 12-well plates were immunolabeled according to previously published protocols [92]. The cells were prefixed (10 min, 37°C) with 0.4 mg/ml bifunctional protein crosslinking reagent DTSP in HBSS [DTSP, 3,3’-dithio-bis(propionic acid *N*-hydroxysuccinimide ester); Hank’s balanced salt solution, Sigma-Aldrich] and preextracted in Tsb [0.5% (w/v) Triton X-100 (Serva) in MTSB] containing 0.4 mg/ml DTSP for 10 min at 37°C, were further extracted in Tsb (5 min, 37°C) and fixed with 4% (w/v) paraformaldehyde (Sevra) in MTSB (15 min, 37°C). After incubation with 0.1 M glycine, the cells were blocked with 1% (w/v) bovine serum albumin (both from Sigma-Aldrich) in Tris-buffered saline, pH 7.6 (BSA-TBS), 2 × 5 min. Mouse monoclonal anti-vimentin antibody (clone V9; Sigma-Aldrich) in BSA-TBS (1:200) was used for a 45-min incubation at 37°C in a moist chamber. After being rinsed thoroughly with BSA-TBS (3 × 5 min), the cells were incubated with goat anti-mouse IgG-TRITC (Sigma-Aldrich) (1:85) or goat anti-mouse IgG-BODIPY (Molecular Probes) in BSA-TBS (1:200) in the same conditions. After washing with BSA-TBS and PBS, the coverslips were counterstained with DAPI (diaminophenyl indole; Sigma-Aldrich), washed 3 × 5 min with PBS and, finally, mounted with Aqua Poly/Mount (Polysciences).

#### G-actin labeling

G-actin fluorescent staining was performed according to two protocols, i.e. using DNase I and vitamin D-binding protein.

In the first case, permeabilization and fixation steps were identical to those described for vimentin staining. After incubation with 0.1 M glycine in DPBS (5 min, RT) and washing with DPBS (2 × 5 min), the cells were incubated with 2 μM phalloidin (Sigma-Aldrich) in DPBS and, subsequently, with DNase I conjugated to Alexa Fluor 488 (Molecular Probes) in DPBS (1:50, 20 min, in the dark). The coverslips were then washed with PBS, stained with DAPI, washed again (3 × 5 min) and mounted on glass slides with Aqua Poly/Mount (Polysciences).

In the second of the above-mentioned methods for indirect staining of non-filamentous actin, previously described procedures were employed, with some slight modifications [93-95]. Coverslips were washed with PBS and fixed with 3.7% (w/v) paraformaldehyde (Serva) in PBS (20 min, RT). Following washing with PBS (3 × 5 min), a 10-min incubation (RT) in PBS with the addition of 1 mg/ml NaBH_4_ (Sigma-Aldrich) was performed in order to reduce free aldehyde groups. Then, the cells were rinsed in PBS (3 × 5 min), extracted in 0.1% (w/v) Triton X-100 in PBS, and further incubated with PBS-TB [0.1% (w/v) Triton X-100/0.2% (w/v) BSA in PBS] for 10 min. Incubation steps aiming at G-actin visualization were as follows: 10 μg/ml vitamin D-binding protein (DBP, Calbiochem) in PBS-TB (60 min, RT), goat polyclonal anti-DBP IgG (Santa Cruz Biotechnology) in PBS-TB (1:50, 60 min, RT), Alexa Fluor 488-conjugated donkey anti-goat IgG (Molecular Probes; 1:100, 60 min, 37°C in a moist chamber). All these incubations were split and followed by rinsing in PBS-TB, 3 × 5 min. Finally, the coverslips were washed twice with PBS, counterstained with DAPI, washed with PBS (3 × 5 min) and subsequently mounted with Aqua Poly/Mount (Polysciences).

#### Cyclin D1 labeling

Cells were briefly washed with PBS, fixed in 4% paraformaldehyde (15 min, RT) and then washed with PBS (3 × 5 min). After that, the cells were incubated in permeabilization solution (0.1% Triton X-100 in PBS, 5 min) and blocked with 1% BSA (30 min). Then, an incubation with mouse monoclonal cyclin D1 antibody (Sigma-Aldrich) (60 min, RT, moist chamber) followed, the cells were washed three times with PBS and incubated with Alexa Fluor 488® goat anti-mouse IgG (Molecular Probes) (45 min, RT). Nuclear staining was performed using DAPI (Sigma-Aldrich). Finally, the cells were washed with PBS and mounted on slides in Aqua Poly/Mount (Polysciences).

#### p21^Waf1/Cip1/Sdi1^labeling

The procedure of prefixation, fixation and blocking was like in the above-described protocol for vimentin. Incubation with mouse monoclonal anti-p21 antibody (sc-817; Santa Cruz Biotechnology) in BSA-TBS (1:100) was performed in a moist chamber for 60 min, RT. Then the slides were rinsed with BSA-TBS (3 × 5 min) and incubated with a secondary antibody (Alexa Fluor® 488 goat anti-mouse IgG conjugate from Molecular Probes) in BSA-TBS (1:100) in the same conditions. The final steps were also as described above.

### SAHF determination and nuclear morphology evaluation

The estimation of SAHF foci formation was performed by counting the DAPI-stained nuclei with the typically distinguished morphology of clearly separated condensations, enclosed within intact nuclear envelope (microscopic observations). For each sample/concentration, at least 500 cells were taken into account for each repeat of the experiment. Other morphological features were also considered for the quantification of nuclear changes after the treatment: the cells with enlarged nuclei, the cells with apoptotic bodies, the cells with nuclei of a regular size but presenting abnormal morphology and the cells with regular nuclei.

An Eclipse E800 microscope and NIS Elements AR imaging software (both from Nikon) were employed to obtain and analyze documentation.

### Flow cytometric analysis of intracellular vimentin

Cells grown on 6-well plates were trypsinized, washed with PBS, centrifuged (5 min, 300 × g) and suspended at a final concentration of 1–2 × 10^6^ cells/ml in 1 ml PBS supplemented with 100 μl of formaldehyde (Polysciences). Samples were incubated for 15 min in the dark and centrifuged (5 min, 300 × g), the resulting pellet was subsequently permeabilized in 2 ml of ice-cold 50% (v/v) methanol (JT Baker). After a 15-min incubation on ice, the cells were washed twice with cold PBS and resuspended in 100 μl of PBS. The cell suspensions were then transferred into flow cytometric tubes containing 20 μl of PE-conjugated mouse monoclonal anti-vimentin IgG_1_ (clone V9), or normal mouse IgG_1_-PE as an isotype control (Santa Cruz Biotechnology). After a 30-min incubation on ice, the cells were washed with PBS, centrifuged for 5 min at 500 × g to remove residues of antibody, and resuspended in 200 μl of PBS for flow cytometric analysis on FACScan (Becton-Dickinson). The percentage of vimentin-positive cells, as well as the mean fluorescence intensity of the cell population was established using CellQuest software (Becton-Dickinson).

### Quantitative immunofluorescence for nuclear G-actin measurements

In order to prevent contamination by cytoplasmic actin, nuclear G-actin was analyzed after isolation of nuclei. Following trypsinization, washing with PBS and centrifugation (5 min, 300xg), 2 ml of homogenizing buffer [50 mM Tris–HCl, pH 7.5; 0.3 M sucrose; 15 mM CaCl_2_; 25 mM MgCl_2_; 10 mM 2-mercaptoethanol (all from Sigma-Aldrich); 0.5% (v/v) Nonidet P40 Substitute] were added to each sample. After homogenization on ice, the suspensions were centrifuged (10 min, 1000 × g) and the supernatant was decanted. The pellet was suspended in 1 ml of homogenizing buffer and gently stratified on top of the buffer for nuclei purification [10 mM Tris–HCl; 0.3 M sucrose; 25 mM MgCl_2_; 25 mM KCl; 10 mM 2-mercaptoethanol (all from Sigma-Aldrich); 41% (v/v) glycerol (Roth)] in fresh tubes (8 ml). The nuclei obtained by centrifugation (10 min, 1000xg) were washed with PBS, fixed in 3.7% (w/v) paraformaldehyde (Sevra), and finally cytocentrifuged on glass slides. Fluorescent staining was performed with vitamin D-binding protein according to the above-mentioned procedure. Digital images from fluorescence microscopy (Eclipse E800; Nikon) were imported to ImageJ software (National Institutes of Health) and converted to grayscale (8-bit). G-actin fluorescence was colocalized with DAPI and subtraction by the nucleus area was performed. Mean fluorescence intensity was presented as the mean grey value, which is the average grey value for all pixels within the indicated area.

### Transmission electron microscopy (TEM)

In order to perform an ultrastructural analysis, cells grown on 6-well plates were trypsinized and fixed with 2.5% (v/v) glutaraldehyde (Merck) in 0.1 M sodium cacodylate (Fluka), pH 7.4 for 30 min at RT. Following washing in cacodylate buffer, the addition of bovine thrombin (Biomed) and fibrinogen (Fluka) resulted in fibrin clot formation, in which cells were entrapped. Then, the samples were postfixed with 2% (w/v) OsO_4_ (Serva) in 0.1 M cacodylate buffer for 1 h at room temperature, passed through a series of ethanol and acetone solutions, and finally embedded in Epon 812 (Roth). Ultrathin sections were prepared, stained with uranyl acetate/lead citrate (Fluka) and examined with a transmission electron microscope (JEM-100CX; JEOL).

### Statistical analysis

Statistical software packages (StatSoft, Tulsa, OK; GraphPad Prism, San Diego, CA) were employed for our analyses. The results obtained were compared using the following tests: the non-parametric Mann–Whitney U test, the Duncan test (for more than 10 repeats, homogeneous variance), as well as the Cochran–Cox test (for non-homogeneous variance). Statistical significance was determined at P<0.05, unless otherwise stated.

## Competing interests

The authors declare that they have no competing interests.

## Authors’ contributions

AL has made substantial contributions to conception and design of the study, acquisition, analysis and interpretation of all the data presented hereby and drafted the manuscript. LG carried out flow cytomertic analyses (Annexin V assay and Flow cytometric analysis of intracellular vimentin), participating in acquisition, analysis and interpretation of the above-mentioned experiments. AH-B carried out flow cytomertic analyses (Cell cycle analysis and TUNEL assay), participating in acquisition, analysis and interpretation of the above-mentioned experiments. AZ was responsible for immunofluorescence protocol of cyclin D1 staining, analysis and interpretation of the experiment. AG has given substantial contributions to conception and topic of the study, has been involved in statistical analyses, interpretation of the results, especially from TEM, and preparation of the manuscript. All authors read and approved the final manuscript.
